# Proteomic Profiling of Colon Cancer Tissues: Discovery of New Candidate Biomarkers

**DOI:** 10.3390/ijms21093096

**Published:** 2020-04-28

**Authors:** Miriam Buttacavoli, Nadia Ninfa Albanese, Elena Roz, Ida Pucci-Minafra, Salvatore Feo, Patrizia Cancemi

**Affiliations:** 1 Department of Biological Chemical and Pharmaceutical Sciences and Technologies (STEBICEF), University of Palermo, Viale delle Scienze, Parco d’Orleans, Building 16, 90128 Palermo, Italy; 2 Experimental Center of Onco Biology (COBS), Via San Lorenzo Colli, 312, 90145 Palermo, Italy; 3 La Maddalena Hospital III Level Oncological Department, Via San Lorenzo Colli, 312, 90145 Palermo, Italy

**Keywords:** colon cancer, proteomic profiling, pathway analysis, transgelin, TAGL

## Abstract

Colon cancer is an aggressive tumor form with a poor prognosis. This study reports a comparative proteomic analysis performed by using two-dimensional differential in-gel electrophoresis (2D-DIGE) between 26 pooled colon cancer surgical tissues and adjacent non-tumoral tissues, to identify potential target proteins correlated with carcinogenesis. The DAVID functional classification tool revealed that most of the differentially regulated proteins, acting both intracellularly and extracellularly, concur across multiple cancer steps. The identified protein classes include proteins involved in cell proliferation, apoptosis, metabolic pathways, oxidative stress, cell motility, Ras signal transduction, and cytoskeleton. Interestingly, networks and pathways analysis showed that the identified proteins could be biologically inter-connected to the tumor-host microenvironment, including innate immune response, platelet and neutrophil degranulation, and hemostasis. Finally, transgelin (TAGL), here identified for the first time with four different protein species, collectively down-regulated in colon cancer tissues, emerged as a top-ranked biomarker for colorectal cancer (CRC). In conclusion, our findings revealed a different proteomic profiling in colon cancer tissues characterized by the deregulation of specific pathways involved in hallmarks of cancer. All of these proteins may represent promising novel colon cancer biomarkers and potential therapeutic targets, if validated in larger cohorts of patients.

## 1. Introduction

Colorectal cancer (CRC) is an aggressive type of tumor and a leading cause of cancer death worldwide [1]. CRC is considered a multifactorial disease: an important role is attributed to the impact of environmental factors on a genetically prone background [2]. Although genetic predisposition is considered an important factor in colorectal carcinogenesis, more than 80% of CRC occurs in the absence of a family history [3,4]. The prognosis of CRC is quite poor [5], with around a 60–65% five-year survival rate. Unfortunately, the survival rate drops dramatically when metastases are found at diagnosis [6,7]. Actually, metastatic dissemination of primary tumors is responsible for 90% of CRC deaths. Pathological staging represents the gold standard for prognosis, although it frequently fails to accurately predict recurrence in patients undergoing curative surgery for locally advanced CRC [8]. In addition, tumors with similar histopathological appearances often manifest significantly different clinical behavior. A large number of CRC patients relapse after complete surgical resection: the most common site of recurrence in CRC is the liver [9]. Other than this, it is critical to understand the molecular heterogeneity associated with different outcomes between individual patients [10], and tumor biology is still mostly unclear. Although several genes have been directly implicated in the etiology of colorectal cancer, and some tumor-intrinsic molecular mechanisms controlling colorectal carcinogenesis have been identified, novel diagnostic and prognostic tools as well as novel therapeutic strategies are still needed to prevent colon cancer progression. This scenario emphasizes the need to identify multiparametric biological markers for more accurate cancer detection and management. The proteomic approach, based on two-dimensional electrophoresis (2-DE) coupled with mass spectrometry (MS), represents one of the most promising techniques for the identification of protein species (differentially expressed and/or post-translationally modified) related to malignancy and useful as potential biomarkers [11]. A valid and potentially valuable strategy in proteomics workflow is the pooling of samples to reduce the biological variance between individual samples [12,13,14]. The underlying assumption is that the measurements recorded on the pool are equal to the average of the measurements on the individual samples.

Our previous studies successfully applied the proteomic approach to discover biomarkers in breast cancer both in vitro [15,16,17,18,19], and in clinical specimens [20,21,22,23,24,25,26,27]. Here, we describe the results of a proteomic survey in 52 human CRC clinical specimens from 26 patients to identify a specific panel of molecular markers correlated with colon carcinogenesis. A comparative proteomic analysis of pooled colon cancer tissues (CCT) paired with adjacent non-tumoral mucosa (NAT) from each patient (*n* = 26) was performed. A number of proteins, associated with well-established cancer hallmarks such as metabolic shift, cell proliferation, evading of cell death and oxidative stress, were up-regulated or down-regulated, and collectively represent targets for further investigation. Moreover, the enrichment analysis of cellular components showed that most of the identified proteins have both intracellular (cytoplasm, lysosomes, mitochondria) and extracellular roles (exosomes, extracellular matrix). Networks and pathways analysis revealed that differentially expressed proteins could be biologically inter-connected to the innate immune response, platelet and neutrophil degranulation and hemostasis, consolidating the importance of the tumor microenvironment and the tumor–host interaction in colon cancer. Interestingly, four different protein species of the TAGL, a 23 kDa actin-binding protein encoded by the TAGLN gene, were identified as top-ranked down-regulated proteins, and further investigated as possible biomarkers for CRC. Altogether, the data of this study contribute to the identification of a more precise colon cancer proteomic profile, which could be used by pathologists to improve patients’ management and care. 

## 2. Results

### 2.1. Pooling of Samples for Proteomics Experiments

This study included 26 patients with histological diagnosis of primary CRC, who underwent surgery without any previous anticancer treatment (chemotherapy or radiotherapy). All other clinical parameters were blinded during the analyses. For each patient, the cancer tissue (CT) and the adjacent healthy tissue (NT) were available. Prior pooling, qualitative and quantitative checks of all the protein extracts from CT and NT were carried out by SDS-PAGE analysis. Although good protein extraction of all the analyzed samples was obtained, a consistent variability was observed both within the CT and NT paired tissue and between different patients (Figure S1). Two pools of colon tumor tissues (CTT) and non-tumoral adjacent tissues (NAT) were generated, respectively, by mixing equal protein concentrations of individual samples.

### 2.2. Differential Proteomic Profile in Colon Cancer Versus Normal Adjacent Tissue

**Figure 1:**
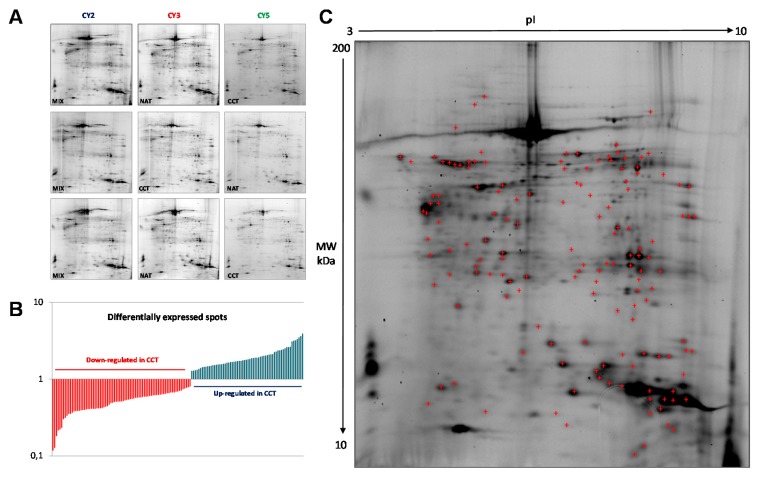
2D-DIGE of pooled colon cancer tissues and non-tumoral adjacent tissues (*n* = 26). (**A**) Miniatures of the 2D-DIGE from NAT and CCT labeled with Cy3 and Cy5 fluorophores and internal standard (equally mixed samples of NAT and CCT), labeled with Cy2. (**B**) Histogram of the 143 differentially regulated spots, up- and down-regulated in CCT versus NAT, selected on the basis of the set threshold (fold change ≥1.2, *p* < 0.05). (**C**) Representative 2D-DIGE of pooled normal adjacent tissues where the identified protein spots are marked. Proteins were focused on IPG strips 18 cm long with a pH range of 3–10. SDS-PAGE was performed on a polyacrylamide gel gradient of 9–16%.

In order to identify colon-cancer-related proteins, a proteomic analysis was performed on both CCT and NAT pools, to minimize the individual variability not associated with cancer. Protein abundance profiles for CCT and NAT (*n* = 26) were compared using 2D-DIGE technology (Figure 1A). After Image Master 2D Platinum analyses of the fluorescent gel images, a total of 2122 spots were matched across all gels, which were run in triplicate. Gel-to-gel matching made it possible to perform statistical analysis of changes in protein abundance between the pooled samples, referring to the internal standard. The differential analysis revealed quantitative changes of 143 spots in CCT versus NAT, with a statistical variance within the 95th confidence level (*p* < 0.05) and 1.2-fold change. Among the differentially expressed spots, 64 and 79 were up- and down-regulated, respectively, in CCT (Figure 1B). A total of 139 protein spots were manually picked from the preparative gels for tryptic digestion and mass spectrometry (MALDI TOF-MS/MS) analyses. Proteins were identified through their peptide mass spectrum matching and by interrogating the Swiss-Prot database. A total of 107 protein spots were successfully identified (Figure 1C, Table S1), corresponding to 61 proteins, since several ones were found in multiple spots as different protein species. When multiple proteins were identified in a single spot, only those exhibiting the number of covering peptides and coverage scores over the cut-off values, together with calculated values of p*I* and molecular weight (MW) closest to the theoretical ones, were considered. 

### 2.3. Functional Classification and Biological Network Analysis

**Figure 2:**
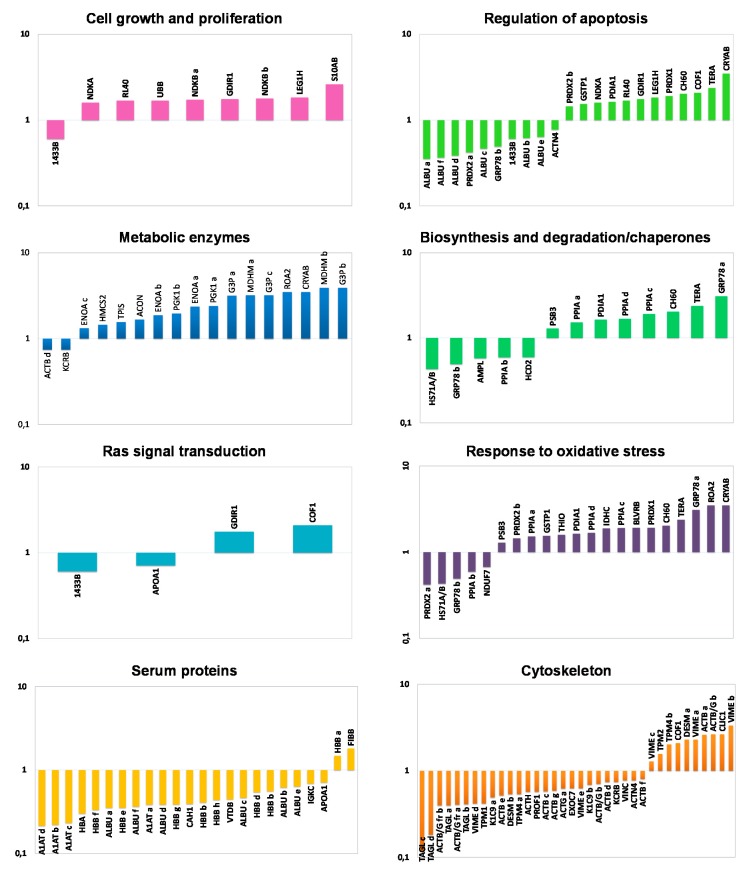
Functional classification of differentially expressed proteins. Histograms of differentially identified proteins, sorted into functional classes according to DAVID functional annotation database classification, were plotted as logarithm of their fold change (CCT versus NAT).

Proteins identified as differentially expressed between NAT and CCT were functionally classified by using the DAVID functional annotation database and grouped into eight functional categories, namely: cell growth and proliferation, regulation of apoptosis, metabolic enzymes, biosynthesis and degradation/chaperones, Ras signal transduction, response to oxidative stress, serum proteins and cytoskeleton. In consideration of the multiple roles often played by many proteins, some of them have been clustered into different functional classes. Figure 2 shows the histograms of the identified proteins sorted into functional classes and plotted as logarithm of their fold change (CCT versus NAT). Interestingly, all the functional categories were related to carcinogenetic processes. As expected, the cluster of metabolic enzymes, cell growth and proliferation as well as the proteins involved in oxidative stress were collectively up-regulated in CCT. Specific proteins or isoforms were found both up- and down-regulated in cancer tissues concerning the clusters of Ras protein signal transduction, biosynthesis and degradation/chaperones and regulation of apoptosis, while the serum protein cluster was collectively down-regulated in CCT. 

**Figure 3:**
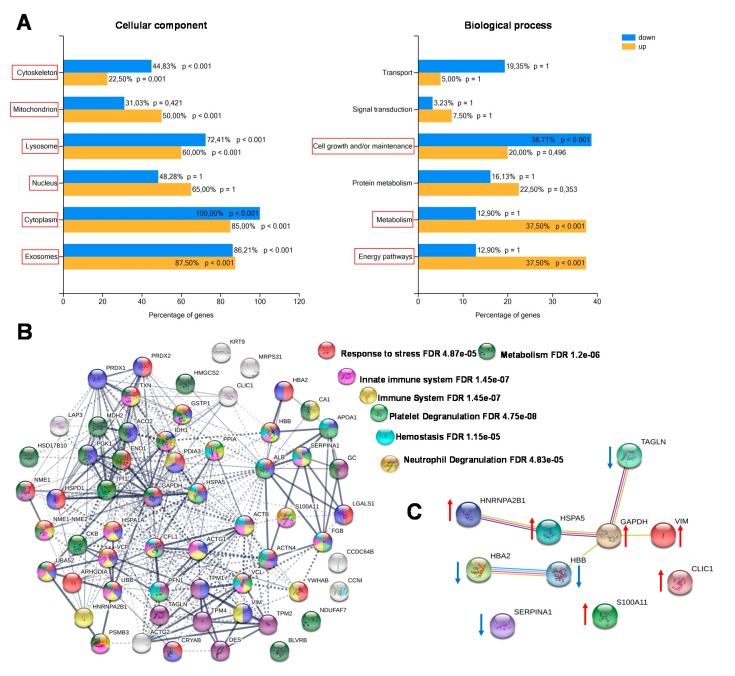
Enrichment and biological network analysis. (**A**) Enrichment analysis of cellular components and biological processes of differentially regulated proteins assessed by Fun Rich. For each biological process and cellular component, the percentage of proteins and the *p* value (Benjamini–Hochberg correction) are indicated. Statistically significant classes are highlighted with red squares. (**B**) Protein–protein interactions and biological process networks generated by the STRING tool. Stronger interactions are represented by thicker lines. Proteins involved in specific biological pathways are highlighted with different colors. (**C**) Functional interaction network of the top-ten differentially expressed proteins between CCT and NAT. Arrows indicate if protein was up- or down-regulated in CCT.

The enrichment analysis of cellular components and biological processes was obtained through Fun Rich software. Most of the identified proteins were recognized as belonging to different cellular components, both intracellular (cytoplasm, nucleus, lysosomes, mitochondria, centrosome) and extracellular (exosomes). The biological processes differentially enriched in CCT compared with NAT were mainly involved in energy pathways, metabolism and cell growth maintenance (Figure 3A). By using the STRING platform, we then analyzed the possible interactions between the identified differentially expressed proteins, in order to reveal networks and pathways able to predict the underlying molecular mechanisms by which colon cancer cells work. The predicted associations included both direct (physical) and indirect (functional) interactions. The interactomic analysis (Figure 3B) showed that the differentially expressed proteins contained more reciprocal interactions than what would be expected for a random set of proteins of similar size (number of edges: 285, expected number of edges: 71; PPI enrichment *p*-value: < 1.0e−16), indicating that they were biologically connected. The biological connection concerned the immune system, in particular the innate immune response; platelet and neutrophil degranulation; and hemostasis. Since the majority of the assigned proteins belong to several of these pathways, further studies will be necessary to determine if the biological connections are really active in colon cancer cells. We also analyzed the top ten differentially expressed proteins between colon cancer and normal adjacent tissues, including those up- and down-regulated (Figure 3C). As a result, seven of them (TAGL, VIM, GAPDH, HSPA5, HBB, HBA2 and HNRNPA2B1) were functionally interconnected, while SERPINA1, S100A11, and CLIC1 were not connect to the obtained network.

### 2.4. TAGL as Possible Biomarker in Colon Cancer

**Figure 4:**
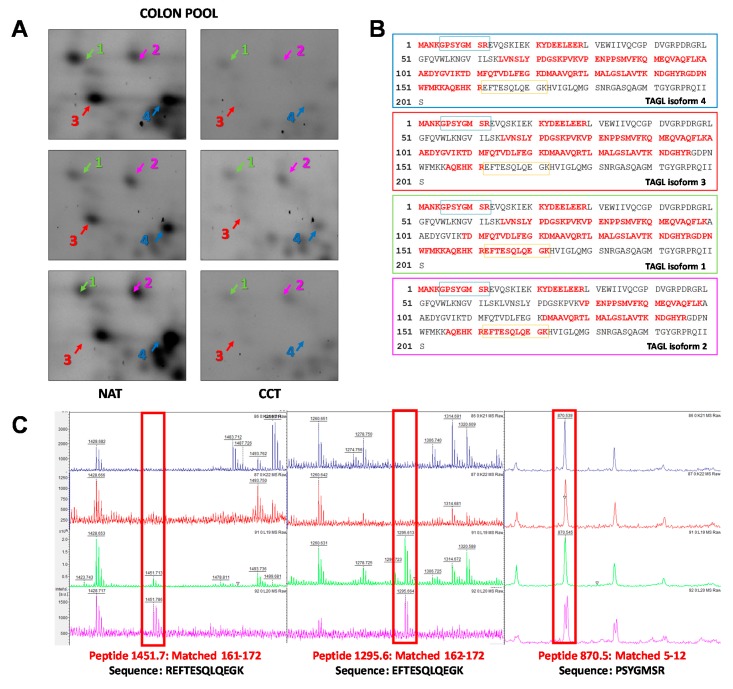
Spectrometric analysis of TAGL protein species. (**A**) Panel showing gel windows from 2D-DIGE gels comprising an area covering a pI/kDa range of 7.2–8.5/30–15 kDa, in which the TAGL protein spots are focused (indicated with different colors by numbers (1–4), starting from the most acidic one), collectively down-regulated in pooled colon cancer tissues. (**B**) Aminoacidic sequence of TAGL showing, for each identified isoform, the sequence coverage of the matched peptides (in red) obtained by MALDI/TOF MS analysis Blue and orange rectangles represent the peptides 5–12 and 162–172 highlighted in red in panel C. (**C**) Details of MALDI/TOF MS spectra showing the presence of specific peaks with specific mass/charge ratios (*m*/*z*) overlapping with the indicated amino acid residues of TAGL. For each protein species, the color of MALDI/TOF MS spectrum correspond to those indicated in panel A.

**Figure 5:**
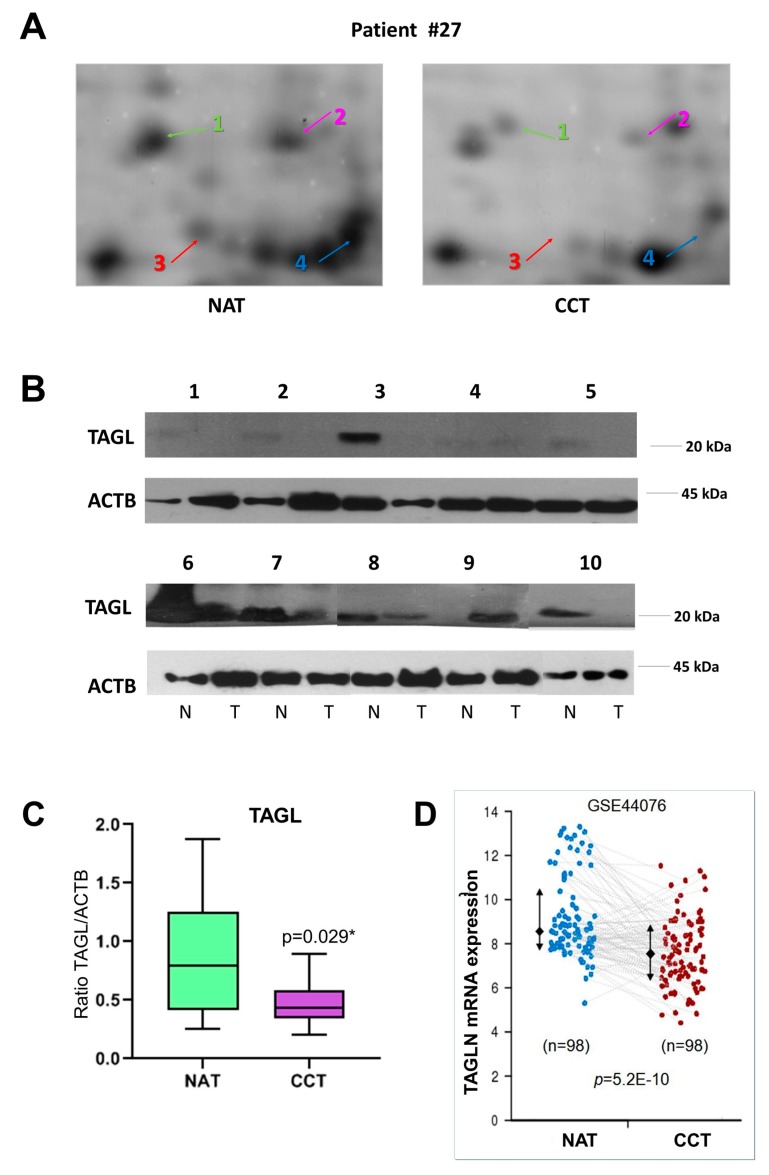
TAGL expression in colon cancer. (**A**) Details of proteomic maps (2D electrophoresis, silver stained) of a colon cancer and normal adjacent tissue derived from a patient not included in the generation of the pool, showing the different TAGL-identified protein species, indicated with different colors by numbers (1–4), starting from the most acidic one. (**B**) Western blot analysis of TAGL abundance in a subset of colon and paired normal tissues (*n* = 10). Actin was used as internal loading control. (**C**) Graphic quantification of western blot results *p* < 0.05 was considered significant and indicated with *. (**D**) Expression levels of the TAGLN gene in 98 colon cancer tissues compared to the corresponding paired normal tissues. Data were extracted from GSE44076 dataset analyzed with the Colonomics data browser (www.colonomics.org).

Among the differentially expressed proteins, TAGL was chosen for further validation, since it is among the top ten modulated proteins, and because four different protein species, collectively down-regulated in colon cancer tissues, were identified in our proteomic investigation. Figure 4A shows gel windows of 2D-gels of CCT and NAT, where the four TAGL protein species are indicated by numbers (1–4), starting from the most acidic one. Two TAGL protein species display a molecular weight (MW) of about 23 kDa, representing the closest expected value of the native protein, while the others display a lower MW, (ca. 19 kDa), likely due to proteolytic cleavage and/or alternative splicing. All spots display different isoelectric points (pI), probably due to post-translational modifications. To provide new insight into the expression of these different protein forms, the MALDI/TOF spectra obtained for each isoform were comparatively analyzed. Figure 4B reports, for each isoform, the sequence coverage (highlighted in red) obtained by the spectrometric analysis. All analyzed protein species start from the N-terminal end, while, when the C-terminal end is taken into account, forms three and four lack sequence coverage compared to forms one and two, suggesting that the lowest MW (protein species three and four) are C-terminal truncated. In particular, as shown in Figure 4C, all recorded spectra display a peak with a mass/charge ratio (*m*/*z*) of 870.5, overlapping with amino acid residues 5–12. In contrast, the peak with *m*/*z* ratio of 1451.7 overlapping with the 161–172 residues, as well as the peak with m/z of 1295.6, overlapping with the 162–172 residues, was present only in the mass spectra of forms one and two. No further information was instead disclosed regarding the differences of pI between different protein species. The proteomic analysis of the colon pool (NAT and CCT) was compared also to that of a breast pool (NAT versus BCT) using 2D-DIGE. The breast pool was generated by mixing equal protein concentrations of individual samples of breast cancer and normal adjacent tissues of 24 patients (*n* = 24). In the colon pool, all TAGL protein species were down-regulated in CCT as compared to NAT, while, in the breast pool, the abundance of TAGL was modest in both healthy and tumor tissues, and no significant differences were detected between NAT and BCT (Figure S2). The differential expression of TAGL between colon cancer and normal adjacent tissues was further investigated by 2D electrophoresis in an independent colon cancer patient not included in the pool. As shown in Figure 5A, TAGL is down-regulated in colon cancer tissue compared to normal adjacent tissue. To confirm modulation of TAGL expression, a western blot analysis of individual specimens (*n* = 20) was performed (the original figures are shown in Figure S3). The obtained results confirmed the down-regulation of TAGL in CCT versus NAT (Figure 5B,C), with the exception of patient nine, where TAGL was detected only in tumoral tissue. Interestingly, TAGL expression was almost variable among the analyzed paired samples, suggesting that TAGL could play important roles also in colon cancer progression. The transcription levels of the TAGLN gene between colon cancer and normal adjacent tissues was also investigated in a larger sample set. Figure 5D shows the expression levels of the TAGLN gene derived from the GSE44076 dataset of Gene Expression Omnibus (GEO) (www.ncbi.nlm.nih.gov/geo/), containing genome-wide gene expression profiles of 98 colon cancer tumors and paired normal adjacent tissues [28]. Consistent with our proteomic results, a significant down-regulation of TAGLN mRNA expression (FC= −1.6; *p*= 5.2 × 10^−10^) was found in colon cancer tissues compared to the corresponding paired normal tissues. 

## 3. Discussion

Recently, significant advances in cancer management have been made, with early diagnosis being one of the most important factors in successful treatment. Aiming to identify proteins involved in biological pathways of colon carcinogenesis, useful for clinical application, a comparative proteomic analysis between pooled specimens of colon cancer and normal adjacent tissues was performed. Sixty-one proteins in 107 spots were identified as differentially expressed (up- and down-regulated) in CCT versus NAT. Functional classification, along with enrichment analysis of biological processes, highlighted significant changes in cancer-related pathways, including cell growth and proliferation, metabolic shift and apoptosis, which represent central cancer hallmarks necessary to understand the multistep process of carcinogenesis. 

Cancer cells sustain proliferative signaling through both the autocrine and paracrine pathways or through overexpressing membrane receptors, resulting in constitutive activation of downstream circuits of signaling pathways [29]. The majority of identified proteins within these classes were up-regulated in pooled colon cancer tissues. 

During tumor progression, cancer cells are able to resist to cell death through the down-regulation of pro-apoptotic factors and sensors of DNA damage, as well as by the overexpression of either anti-apoptotic regulators or survival signals [30]. Within this functional class, the most up-regulated protein was Alpha B-crystallin (CRYAB), a molecular chaperone capable of preventing aggregation and degradation of damaged unfolded proteins resulting from heat shock, radiation and oxidative stress; promoting overall cell survival; and preventing apoptotic events. The expression of CRYAB was significantly associated with poor prognosis in CRC patients [31]. 

Among the metabolic enzymes, those belonging to the glycolytic pathway were collectively up-regulated in cancer tissues. Thus, our findings agree with the anaerobic shift of the metabolism of cancer cells [32], also known as the Warburg effect. This is a common phenomenon during the development of several tumors and seems to be a key step for tumor progression [33].

Within the class of biosynthesis and chaperone proteins, GRP78 was found to be the most up-regulated protein along with an identified isoform which was down-regulated in CCT. GRP78 is a key regulator of endoplasmic reticulum stress with an important role in regulating CRC cell proliferation and apoptotic cell death. Although its overexpression has been correlated with tumor aggressiveness, literature data attribute opposite roles to GRP 78 in colon cancer progression [34,35]. This discrepancy may be due to the existence of different post-translational isoforms, which could enable proteins to perform different functions through interactions with different proteins. 

Interestingly, while the functional classes so far analyzed are involved in generic mechanisms of carcinogenesis, the cluster of proteins involved in Ras-signaling is specifically related to colon cancer genesis, strengthening the obtained results. For example, 14-3-3 protein beta/alpha (1433B) plays important roles in cancer signaling. Collectively, the 14-3-3 proteins are considered tumor suppressors, whose down-regulation has been frequently detected in tumor specimens of many types of cancer [36].

Most proteins involved in responses to oxidative stress were up-regulated, with the exception of PRDX2, GRP78, and PPIA, found as different protein species both up- and down-regulated, increasing the complexity level that each protein isoform could produce within the cell. Oxidative stress is an imbalance ratio between reactive oxygen species (ROS) production and antioxidants and represents a pivotal factor in CRC development and progression. Accumulating evidence highlights that CRC risk factors like smoking and alcohol consumption, as well as chronic inflammation, are involved in ROS production [37,38]. Moreover, dysbiosis, altered structure and function of the gut microbiota, has been associated with the development of CRC through different mechanisms, including generation of reactive metabolites and carcinogens, altered host carbohydrate expression, as well as induction of chronic mucosal inflammation [38]. The sustained inflammatory/oxidative environment leads to the activation of immune and inflammatory pathways in a vicious circle. In turn, a multifactorial network of chemical signals initiate and amplify the recruitment of leukocytes (i.e., neutrophils, monocytes, and eosinophils) lymphocytes, and platelets, promoting a pro-tumorigenic microenvironment [39]. Interactions between immune cells and tumor cells (host–tumor interactions) trigger a new complex signaling cascade. Neutrophils, physically interacting with circulating tumor cells, can promote tumor progression by inducing tumor cell proliferation, stimulating angiogenesis and matrix remodeling, and disabling T cell-dependent antitumor immunity, therefore facilitating the binding of tumor cells to the endothelium [40]. Moreover, neutrophil granule proteins released upon cell activation might play a significant role in promoting the transport and extravasation of circulating tumor cells, thus facilitating their metastatic migration [41]. Tumor cells can also directly interact with platelets, inducing their aggregation and degranulation. Once again, platelet granules determine the establishment of new persistent cross-talk with cancer cells. For instance, most serum vascular endothelial growth factor (VEGF) derives from degranulation of granulocytes and platelets [42]. VEGF is known to promote both vascularization and tumor growth. On the other hand, platelets also contain hemostatic factors that inhibit angiogenesis. The hemostatic system contributes to the development of the malignant phenotype acting at different levels. Accumulating evidence has revealed the role of various components of the coagulation system in different stages of carcinogenesis [43]. All these explanations are consistent with networks and pathway analysis performed with our differentially expressed proteins; indeed, biological connection was found with tumor-host microenvironment, including innate immune response, platelet, neutrophil degranulation and hemostasis, as well as a differential expression of several serum proteins. Collectively, the serum proteins were down-regulated, except for fibrinogen (FIBB), which is an acute phase protein whose concentration differs significantly in response to inflammatory processes. Albumin (ALBU) is an antioxidant protein, which thus may be anticarcinogenic. Accordingly, several studies have reported an increased risk of cancer mortality associated with low serum albumin concentrations [44]. Over the last years, several differences in plasma proteins have also been detected during colorectal cancer progression [45]. Moreover, it is well known that solid tumors are constantly exposed to low oxygen levels due to excessive cellular proliferation, and the oxygenation status is tightly linked to aggressive behaviour, since hypoxia represents the major driving force behind tumor vascularization and invasion [46]. Cancer cells respond differently to hypoxia leading to cell death or cell survival. In the latter case, key cellular responses to hypoxia induce new blood vessel formation, namely angiogenesis. During hypoxia, lower abundance of circulating blood proteins should be evident; on the contrary, during neoangiogenesis, higher abundance of circulating blood proteins in tumor tissues should be detected. These aspects deserve further investigation. The last functional protein class identified in this proteomic study included cytoskeleton proteins. Cytoskeleton represents not only an essential structural support but also a functional system for the cells. It is responsible for cell shape, motility and signaling. Among the identified proteins within this category, vimentin (VIME, three protein species) and chloride intracellular channel 1 (CLIC1) were significantly up-regulated in colon cancer tissues. VIME belongs to the intermediate filament proteins involved in cell attachment, migration, and signaling. Moreover, VIME represents a biomarker for the epithelial to mesenchymal transition (ETM), an important process required for tumor invasion. The overexpression of VIME in CRC was identified as a predictive biomarker for lymph node metastasis and poor prognosis [47]. CLIC1 is a multifunctional protein, displaying ion channel, redox and enzymatic properties, and also capable of acting as a scaffold protein [48]. Several studies have reported CLIC1 to be an overexpressed protein in CRC [49], involved in cell migration and invasion, including more tumor recurrences and shorter patient survival, through the regulation of MMP-2 and MMP-9 [50,51]. Since the colon is primarily responsible for the absorption of water and electrolytes, the up-regulation of CLIC1 as a chloride anion channel could be involved in the deregulation of the extracellular milieu. The most down-regulated protein was TAGL (identified with 4 protein species), a 23 kDa actin-binding protein. TAGL is an actin crosslinking/gelling protein expressed in endothelial cells, smooth muscle cells, fibroblasts and several immune cells. TAGL induces smooth muscle cell differentiation, regulating development and contractile function of these cells. Moreover, it is actively involved in the actin-cytoskeletal rearrangements regulating cell migration, podosome formation, tissue invasion, and matrix remodeling [52,53]. TAGL is associated with a specific sub-population of actin filament bundles, and its depletion increased the capacity of cells to form podosomes, increased the actin dynamics, and enhanced the tumorigenic properties of cells [54,55]. Most studies reported that TAGL acts as a tumor suppressor [56], inhibiting cell migration, suppressing the 92-kDa type IV collagenase (MMP-9) [57], and being down-regulated by the Ras pathway [58] as well as silenced through epigenetic mechanisms, which involve TAGL promoter hypermethylation [59,60]. Moreover, TAGL inhibits cell proliferation and invasion in vitro, and also blocks tumorigenesis in vivo in other cancer types [55]. However, some reports have indicated that TAGL has a pro-tumorigenic role, described as a negative prognostic factor overexpressed in tumor tissues [61,62]. The up-regulation of TAGL was also observed in gastric and pancreatic cancers [63]. Based on these controversial results, the pathological role of TAGL appears to differ between cancer types and could change during tumor progression. Down-regulation of TAGL in colon cancer versus normal adjacent tissues was further validated by Western blot. Our results suggest that TAGL could be a promising and specific biomarker for CRC, suitable for diagnosis and prognosis. The variability of protein abundance levels found between the analyzed paired samples deserves further investigation.

The mass spectrometry analysis showed that two TAGL protein species with the lowest MW (about 19 kDa), were C-terminal truncated. Although the molecular mechanism involved in the formation of these truncated forms is unknown, the functional effects could concern their ability to bind actin. It is reported that the C-terminal portion of TAGL contains an actin-binding domain, namely, calponin-like domain (CLIK23). This domain is responsible for the binding ability of TAGL to actin, whose binding is impaired by the deletion of this domain [64]. Moreover, in the C-terminal end the binding sites for kinases are found. In this regard, in-vitro experiments showed that phosphorylation by protein kinase C decreased the ability of TAGL to bind actin [65]. Thus, it is reasonable to suggest that the two protein species may exert diverse functions into the cell. We think that this is an important contribution to knowledge of TAGL protein species, opening intriguing questions that deserve further investigation. We believe that the results reported here, although they have significant clinical implications, should be taken with caution, due to the limited number of analyzed patients and the fact that stringency in fold change was not considered.

## 4. Materials and Methods

### 4.1. Patients and Tissue Samples 

The study included 27 patients affected by CRC, whose clinical characteristics were blinded during the analyses. All patients underwent surgical removal of the tumor at La Maddalena Hospital, without any cytotoxic treatment prior to surgery. Inclusion criteria were age less than 65 years and no metastases at diagnosis. Tumor specimens (CT) and normal adjacent samples (NT) were snap frozen and stored at −80 °C until protein extraction. Research was carried out in compliance with the Helsinki Declaration with patients’ written consent and with the approval of the Institutional Review Board (N/515/2008) from the La Maddalena Hospital. For validation experiments, a cohort of breast cancer specimens, previously used in proteomic investigations was also used [20,21,22,24,26,27].

### 4.2. Tissues Processing and Pooling Samples

The frozen tissues were homogenized at 4 °C with lysis buffer [30 mM Tris, pH 8.5, 7 M urea, 2 M thiourea, 0.4% *w*/*v* CHAPS, 1% *w*/*v* 1,4-dithioerythritol (DTE)] and incubated under rotation overnight at 4 °C. Tissue lysates were centrifuged several times at 14,000 rpm for 20 min to remove cell debris. Protein concentration was determined by Bradford assay, as already reported [66,67]. 

The pools of colon cancer tissues (*n* = 26) and normal adjacent tissues (*n* = 26) were obtained by combining the same amount in terms of protein concentration (100 µg of total proteins for each sample). Pools of breast cancer tissues (*n* = 24) and normal adjacent tissues (*n* = 24) were also prepared. 

### 4.3. Electrophoresis SDS-PAGE and Western Blotting

Aliquots containing 20 µg of cell lysates from colon cancer tissues and paired non-tumoral adjacent tissues were separated by electrophoresis on 10% or 12% sodium dodecyl sulfate (SDS)–polyacrylamide gels, under reducing conditions. After electrophoresis, gels were stained with Coomassie Brilliant Blue G 250 and de-stained with H_2_O milliQ or transferred into a nitrocellulose membrane and stained with Ponceau S (Sigma Aldrich, St. Louis, MO, USA). Membranes were blocked with 5% milk in T-TBS solution for 1 h at room temperature and then incubated overnight at 4 °C with a mouse monoclonal antibody for TAGL or Actin β by Santa Cruz Biotechnology (Santa Cruz, CA, USA). Following incubation with the mouse peroxidase-linked antibody, the reaction was revealed by the ECL detection system, using high-performance films (Hyperfilm ECL, Amersham, GE Healthcare, UK), as already described [68,69]. The correct protein loading was ascertained by red Ponceau staining and immunoblotting for Actin β. 

### 4.4. 2-D-Differential-In-Gel-Electrophoresis (2D-DIGE)

Fifty µg of each sample were minimally labeled, using the CyDyeTM DIGE minimal labeling kit (GE Healthcare, Sweden), with 400 pmol of CyDyes DIGE Fluors Cy3 and Cy5 and incubated on ice in the dark for 30 min, as already described [70]. An internal standard was generated by combining equal amounts of extracts from all CCT and NAT or breast and colon pool samples and labeled with Cy2. The first dimensional separation was performed using immobilized pH gradient IPG strips (3–10 NL range, 18 cm) onto an IPGphor (GE Healthcare Bio-Sciences, Uppsala Sweden) apparatus (67 kVh, 20 °C). Strips were rehydrated in 8 M urea, 2% CHAPS, 10 mM DTE, and 0.5% carrier ampholytes (Resolyte 3.5–10). The isoelectrofocusing was carried out by linearly increasing voltage from 200 to 3500 V during the first 3 h, after which focusing was continued at 8000 V for 8 h. After focusing, each strip was equilibrated twice with a solution containing 6 M urea, 30% glycerol, 2% SDS, 0.05M Tris-HCl pH 6.8, and 2% DTE for 12 min, to resolubilize proteins and reduce disulphuric bonds. The -SH groups were then blocked by substituting the DTE with 2.5% iodoacetamide in the equilibrating buffer. The focused proteins were then separated on 9–16% linear gradient polyacrylamide gels (SDS-PAGE) using a DALT six (GE Healthcare Bio-Sciences, Uppsala Sweden) apparatus with a constant current of 40 mA/gel at 10 °C. Images were acquired with a Typhoon scanner (GE Healthcare Bio-Sciences, Uppsala Sweden) using specific emission filters. Images were analyzed using DeCyder Differential Analysis Software v7.2 (GE Healthcare Bio-Sciences, Uppsala Sweden). The software calculates normalized intensities (standard abundance) for all spots by comparison with the internal standard, and then an average volume ratio and relative *p*-value by Student’s paired t-test. Only protein spots with 1.2-fold changes in volume after normalization in at least three separate experiments (*p* < 0.05) were considered differentially expressed and selected for further characterization. After the acquisition, each gel was stained with ammoniacal silver nitrate. 

### 4.5. Protein Identification by MALDI-TOF MS

Spots of interest were manually picked, while mass spectrometric sequencing was carried out after in-gel digestion, using sequencing-grade trypsin (20 μg/vial), according to the method of Shevchenko et al. with some modifications, as described elsewhere [22]. The tryptic peptide extracts were dried in a vacuum centrifuge and dissolved in 0.1% trifluoroacetic acid (TFA). Peptide mixtures were desalted by μZip-TipC18 (Millipore, MA, USA). The matrix, R-cyano-4-hydroxycinnamic acid (HCCA), was purchased from Sigma-Aldrich. A saturated solution of HCCA (1 μL) at 2 mg/200 μL in CH_3_CN/H_2_O [50:50 (*v*/*v*)] containing 0.1% TFA was mixed with 1 μL of peptide solution and loaded onto the MALDI target plate and left to dry. A peptide calibration standard was spotted separately onto the MALDI target plate. Mass spectra were obtained using an Ultraflex MALDI-TOF-TOF (Bruker Daltonics, Bremen, Germany) mass spectrometer. Peptide mass fingerprinting was compared to the theoretical masses from the Swiss-Prot or NCBI sequence databases using Mascot (http://www.matrixscience.com/). Typical search parameters were as follows: 50 ppm of mass tolerance, carbamidomethylation of cysteine residues, one missed enzymatic cleavage for trypsin. A minimum of four peptide mass hits was required for a match, methionine residues could be considered in oxidized form, no restriction was placed on the isoelectric point of the protein, and a protein mass range from 5 to 100 kDa was allowed.

### 4.6. Functional Classification and Pathway Analysis

Functional classification of the identified proteins, as well as pathways and network analyses were performed using DAVID, STRING, and FUNRICH on-line tools.

The Database for Annotation, Visualization and Integrated Discovery (DAVID), (http://david.abcc.ncifcrf.gov) provides a comprehensive set of functional annotation tools for investigators to understand biological meaning behind large list of genes [71]. David represents a powerful method to group functionally related genes and terms into a manageable number of biological modules for efficient interpretation of gene lists in a network context. 

STRING (https://string-db.org) is a database of known and predicted protein-protein interactions. The interactions include direct (physical) and indirect (functional) associations; they stem from computational prediction, from knowledge transfer between organisms, and from interactions aggregated from other (primary) databases analysis [72]. 

FunRich (http://www.funrich.org/) is a stand-alone software tool used mainly for functional enrichment and interaction network analysis of genes and proteins and to identify over-represented classes [73]. The functional enrichment analysis tool makes it possible to graphically analyze the results in the form of Venn, bar, column, pie and doughnut charts. 

### 4.7. Statistical Analysis

The relative levels of stained protein spots compared with the internal standard spots were analyzed by the DeCyder Difference In-gel Analysis software module (GE Healthcare). A Student’s *t*-test was used to calculate statistically significant differences between the two groups in relative abundance of individual protein spots among the groups in 2D-DIGE. *p* < 0.05 was considered statistically significant.

## 5. Conclusions

Differential proteomics made it possible to identify several candidate biomarkers for colon cancer. In particular, TAGL, identified as a down-regulated protein (four different protein species) in CCT, emerged as a top-ranked biomarker for CRC. Our finding revealed different proteomic profiling in colon cancer tissues characterized by deregulation of specific pathways involved in hallmarks of cancer. Each of these proteins may represent novel and promising colon cancer biomarkers, which are potential therapeutic targets if validated in larger cohorts of patients. Further investigations are needed to determine whether the proteins identified in this study play any functional roles in colon cancer progression and metastases.

## Supplementary Materials

The following are available online at https://www.mdpi.com/1422-0067/21/9/3096/s1, **Figure S1:** SDS-PAGE (Sodium docecyl sulfate–polyacrylamide gel electrophoresis) of total protein extracts from 26 colon cancer tissues (CT) and paired normal tissues (NT) after staining with Blue Coomassie solution; **Table S1:** List of differentially expressed proteins between CCT and NAT identified by MALDI-TOF/MS. All differences are statistically relevant, with *p* < 0.05. Some proteins were present in different spots, generally represented by post-translational modifications and indicated with alphabetic letters. Spot number, fold change, protein name, gene name, accession number, MASCOT Score, number of matched peptides, sequence coverage (%), theoretical MW and theoretical pI are indicated. **Figure S2:** Details of proteomic maps (2D-DIGE) of colon (**A**) and breast (**B**) pools (both cancer and normal adjacent tissues) showing the different TAGL-identified protein species. **Figure S3:** Original western blots of TAGL (**A**) and ACTB (**B**).
