# Supplementary material for: Proteomic Profiling of Colon Cancer Tissues: Discovery of New Candidate Biomarkers
Source: Int J Mol Sci. 2020 Apr 28;21(9):3096. doi: 10.3390/ijms21093096 (PMC7247674; doi:10.3390/ijms21093096)
Supplement: Supplementary file 1 [file ijms-21-03096-s001.zip › Supplemetary Figures.pdf]

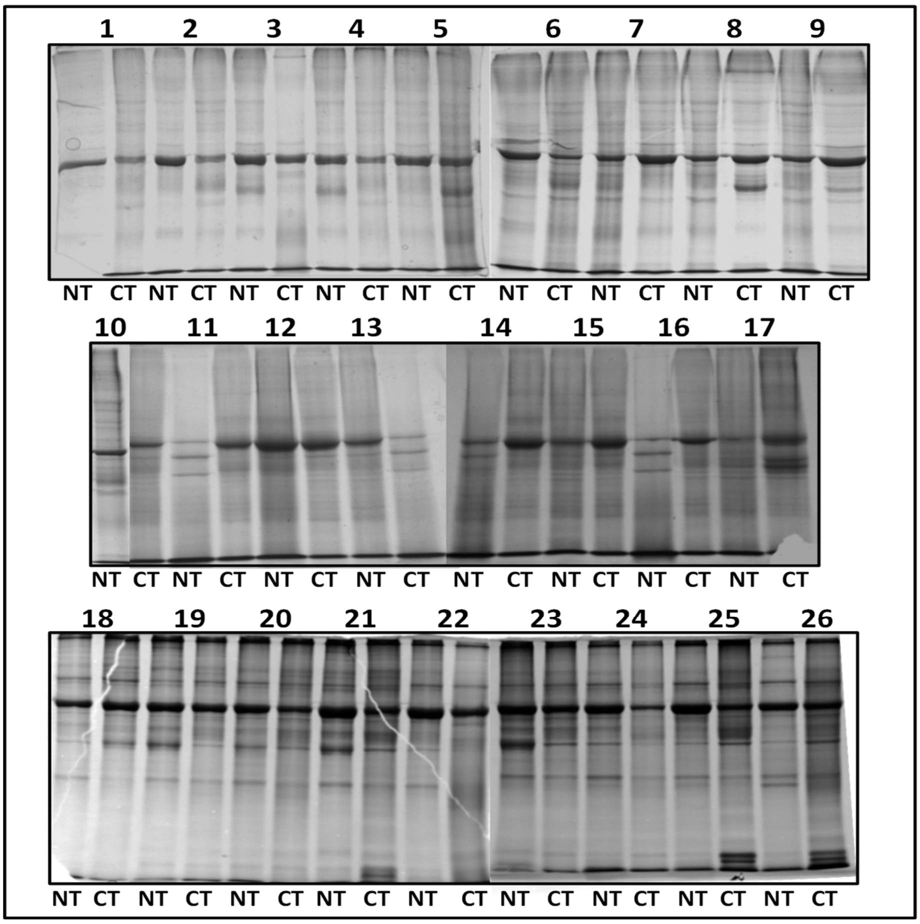

Supplemetary Figure 1: SDS-PAGE (Sodium docecyl sulfate –polyacrilamide-gel electrophoresis) of total protein extracts from 26 colon cancer tissues (CT) and paired normal tissues (NT) after staining with Blue Coomassie.

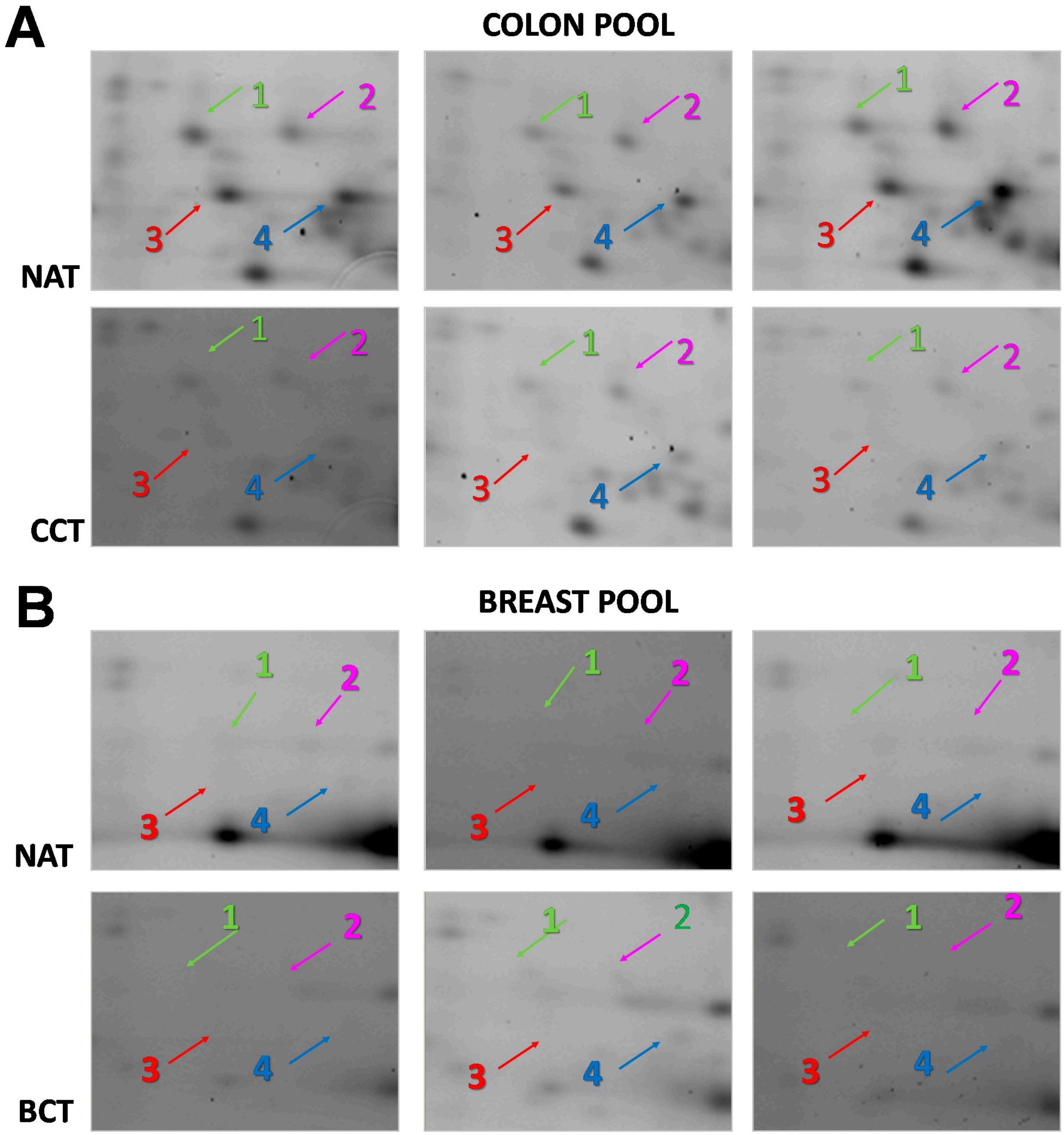

Supplemetary Figure 2: Details of proteomic maps (2D-DIGE) of colon **A)** and breast **B)** pools (both cancer and normal adjacent tissues) showing the different TAGL identified protein species.

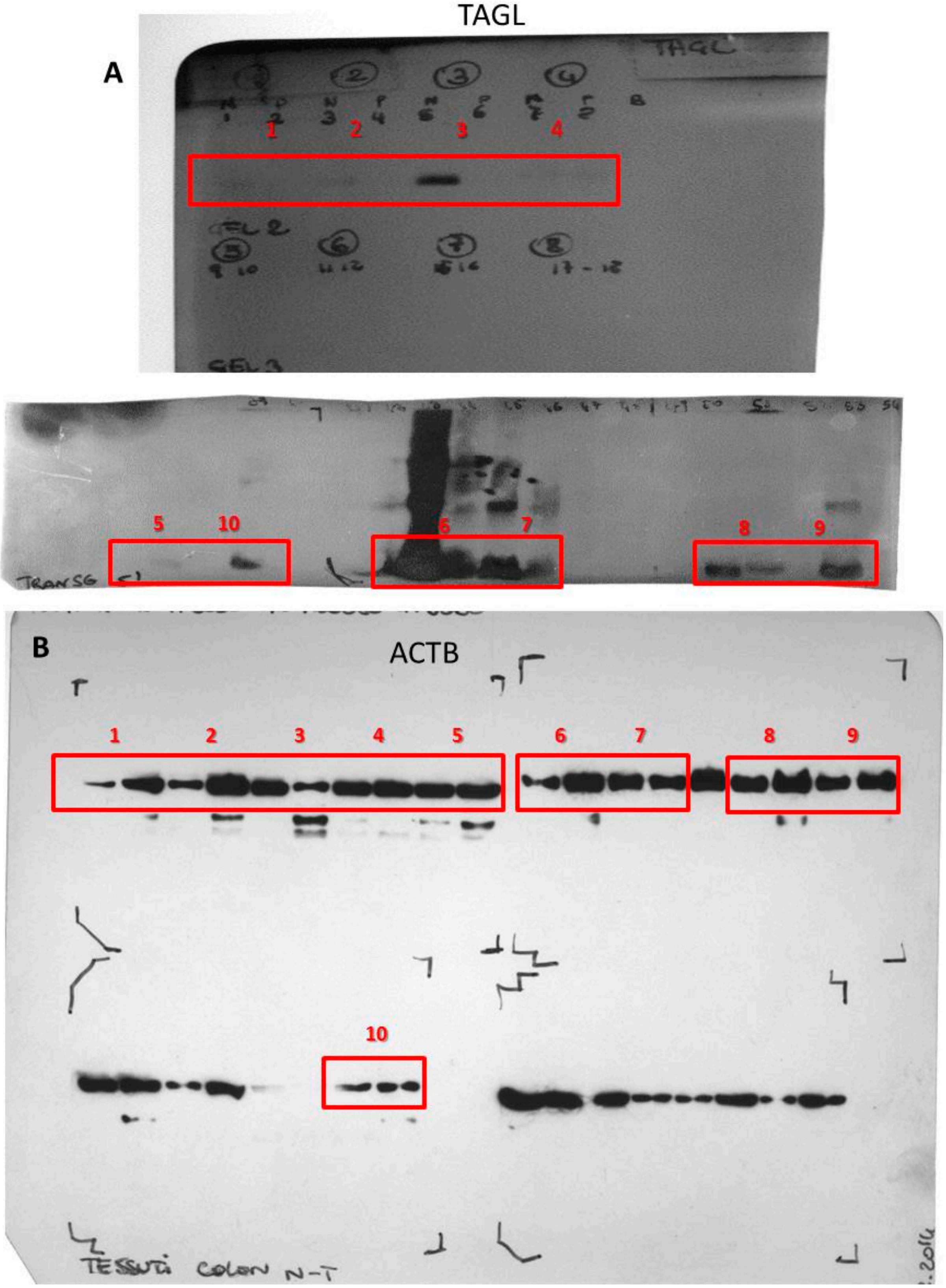

Supplemetary Figure 3: Original western blots of TAGL **A)** and ACTB **B).**
